# Metabolic Aspects of Palladium(II) Potential Anti-Cancer Drugs

**DOI:** 10.3389/fonc.2020.590970

**Published:** 2020-10-12

**Authors:** Tatiana J. Carneiro, Ana S. Martins, M. Paula M. Marques, Ana M. Gil

**Affiliations:** ^1^ Department of Chemistry, Center for Research in Ceramic and Composite Materials (CICECO)—Aveiro Institute of Materials (CICECO/UA), University of Aveiro, Campus Universitário de Santiago, Aveiro, Portugal; ^2^ “Molecular Physical Chemistry” R&D Unit, Department of Chemistry, University of Coimbra, Coimbra, Portugal; ^3^ Department of Life Sciences, University of Coimbra, Coimbra, Portugal

**Keywords:** palladium, complexes, chelates, anti-cancer activity, metabolism

## Abstract

This mini-review reports on the existing knowledge of the metabolic effects of palladium [Pd(II)] complexes with potential anticancer activity, on cell lines and murine models. Most studies have addressed mononuclear Pd(II) complexes, although increasing interest has been noted in bidentate complexes, as polynuclear structures. In addition, the majority of records have reported *in vitro* studies on cancer cell lines, some including the impact on healthy cells, as potentially informative in relation to side effects. Generally, these studies address metabolic effects related to the mechanisms of induced cell death and antioxidant defense, often involving the measurement of gene and protein expression patterns, and evaluation of the levels of reactive oxygen species or specific metabolites, such as ATP and glutathione, in relation to mitochondrial respiration and antioxidant mechanisms. An important tendency is noted toward the use of more untargeted approaches, such as the use of omic sciences *e.g.*, proteomics and metabolomics. In the discussion section of this mini-review, the developments carried out so far are summarized and suggestions of possible future developments are advanced, aiming at recognizing that metabolites and metabolic pathways make up an important part of cell response and adaptation to therapeutic agents, their further study potentially contributing valuably for a more complete understanding of processes such as biotoxicity or development of drug resistance.

## Introduction

The discovery of the important antitumor activity of cisplatin [*cis*-diamminedichloroplatinum(II), *cis*-Pt(NH_3_)_2_Cl_2_, cDDP] (1), a complex that targets DNA resulting in the formation of intra- and inter-strand DNA cross-links and triggering apoptotic cell death, led to it becoming the first successful metal-based anticancer drug (2). This prompted much interest in inorganic compounds as potential antineoplastic agents, platinum [Pt(II)] complexes remaining some of the most efficient chemotherapeutic drugs used in the clinic. However, such compounds are typically associated to serious systemic toxicity and acquired resistance (mainly *via* interaction with glutathione and metallothioneins) (3). Therefore, other metal complexes have been tested, namely containing ruthenium (4, 5), titanium (6), gold (7), and palladium, Pd (8–11). Pd(II) compounds have attracted much attention due to the similarity of the metal center to Pt(II) (electronic structure and coordination chemistry) (12, 13). Such complexes have shown favorable cytotoxic activity despite their higher lability compared to Pt(II) analogues (10, 11). *In vivo* stability of Pd(II) antitumor complexes relies on strongly coordinating ligands (*e.g*., dithiocarbamates, polydentate linear amines) and reasonably nonlabile leaving group(s) (13, 14). Both mono- and polynuclear Pd-complexes (*i.e.*, containing one or more than one metal centers) have been synthesized and characterized (8–11, 13), along with heterometallic Pt/Pd-complexes (di- and trinuclear), in order to improve antineoplastic response and patient survival (15). In contrast to mononuclear metal agents, which can only bind (covalently) to one or two sites at their main pharmacological target (DNA bases), polynuclear compounds may interact with DNA at several sites on the double helix (*via* short or long range interactions depending on the nature of the ligands), inducing more severe and less repairable damage. In addition, development of polynuclear agents is based on the assumption that they may lead to interactions with multiple biological targets, simultaneously (*e.g.*, sulfur-donor biomolecules, thioethers, thiols), thus restricting the induced deleterious side effects.

The Pd(II) complexes tested so far in relation to their antitumor activity have, mostly, been characterized in terms of cytotoxic and antiproliferative properties toward different tumor cell lines (15, 16), as well as to their DNA- or protein-binding abilities (17, 18). The knowledge of the ability of metal complexes and, in particular, Pd(II) complexes, to disrupt and induce adaptations in cell/organism metabolism is still scarce, in spite of its importance for the evaluation of the more global effects of these potential new antineoplastic agents. In this context, further and untargeted metabolic studies are of great value to unveil new disruptions in cellular metabolism resulting from exposure to complexes. Indeed, increasing interest is noted in further understanding the metabolic response of tumoral (and occasionally healthy) cell lines or tissues to drugs, both *in vitro* and *in vivo*. In this mini-review, the specific metabolic effects of Pd(II) complexes will be addressed, noting the complexes´ impact on different genes, proteins or metabolite levels, subsequently interpreted as related to specific deviant metabolic pathways. Reports solely based on typical cytotoxicity/cell proliferative actions or DNA and protein binding are not included in this review, which intentionally gathers only the studies that include some reference to metabolic effects.

## *In Vitro* Studies of the Metabolic Impact Of Pd(Ii) Complexes

**Table 1** lists the studies that report the impact of mono- and polynuclear Pd(II) complexes on some aspect of *in vitro* or *in vivo* metabolism, while the chemical structures of the corresponding Pd(II) complexes are shown in **Table S1** (to guide the reader through the structural characteristics of these compounds). It becomes clear that most of the reported studies have addressed mononuclear Pd(II) complexes, often using cisplatin as a reference, and have been conducted in *in vitro* conditions. However, a more recent interest is noted in binuclear Pd(II) complexes, while some of the existing reports also describe comparison of results obtained *in vitro* with those registered *in vivo* in murine models. The text below is, when possible, organized chronologically, for each type of palladium complex investigated.

**Table 1:** List of metabolism-related studies of Pd(II) complexes tested *in vitro* or *in vivo*.

	Pd(II) complexes and ligands (L)		Cell line/animal model	Main metabolic observations	Ref.
***In vitro* studies**					
**Mononuclear**	[(bipy)Pd(Pcurc)][CF_3_SO_3_]; L: pure curcumin (Pcurc), 4,4′-dinonyl-2,2′-bipyridine (bipy)	LnCaP, PC3, DU145 (prostate cancer)		• Apoptosis (caspase-3 activation) • ↑ ROS levels (and ↓GSH), JNK phosphorylation, GSTp1 downregulation • Mitochondrial membrane depolarization, upregulated Bax, downregulated Bcl-2 proteins • Hindered PARP activation, no effect on DNA (contrary to Pcurc alone)	(19)
	[Pd(L)Cl]; L: acyclic tridentate quinoline-2-carboxaldehyde-2-pyridylhydrazone	PC3 (prostate cancer)		• Apoptosis (caspase-3 activation) • G2/M phase cell cycle arrest, cell growth inhibition • Mitochondrial pathway triggered, cytochrome *c* release, higher caspase-3 activity	(20)
	[Pd(sac)(terpy)](sac)•4H_2_O; L: saccharinate (sac), 2,2’:6’,2’’-terpyridine (terpy)	MCF-7, MDA-MB-231 (breast cancer) (comparison to *in vivo*)		• ↑ expression of DR4/DR5 cell death genes, DR5 protein • Possible anti-invasive activity by prevention of tubule formation in MDA-MB-231 (metastatic) • Apoptosis only in MCF-7 (non-metastatic)	(21)
	Pd(MCO)_2_ (compared to Pt(MCO)_2_ and cDDP); L: 2-cyano-2-isonitroso-*N-*morpholylacetamide	HeLa (cervical cancer)		• Unspecified different metabolic impact compared to cDDP family, probably averting negative side effects	(22)
	[Pd(acac)_2_] (compared to cDDP); L: bisacetylacetonate (acac)	H460 (non-small-cell lung cancer) (more lines in other studies and comparison to *in vivo*)		• Apoptosis, *via* ER stress, with CHOP upregulation • ↓ Ca^2+^ levels, ↑ misfolded protein in ER • Upregulation of IRE1 signaling, caspases activation	(23)
	Pd(diethyl dithiocarbamate)_2_ (compared to Pt(II) analogue and other symmetrical Pt(II) and Ni(II) complexes); L: diethyl dithiocarbamate	*K562 (leukemia)*		• Cytosolic antioxidant defense enzymes (GST, GPX, PTK, *CAT)* inhibition pattern, dependent on metal-complex • Pd complex: good antioxidant and antitumor activity	(24)
	Pd(sac)(terpy)](sac)•4H_2_O; L: saccharinate (sac), 2,2’:6’,2’’-terpyridine (terpy)	MDA-MB-231 (breast cancer), A549 (lung cancer), HeLa (cervical cancer)		• MDA-MB-231: 30 proteins with altered expression levels and impacting on multiple pathways, including energy metabolism, double strand break repair mechanisms, membrane trafficking, protein degradation and apoptosis • ↑ ROS levels in all cell lines	(25)
	[Pd(L^1^)_2_], [Pd(L^2^)_2_], [Pd(L^3^)_2_], [Pd(L^4^)_2_] (compared to cDDP); L: 2-(arylazo)phenol with different R and R´: L^1^: R, H; R’=CH_3_, L^2^: R, CH_3_, H; R’=H, L^3^: R, CH_2_CH_3_; R’=H, L^4^: R, (CH_2_) _2_CH_3_; R’=H	A549 (lung cancer), HeLa (cervical cancer), PA-1 (teratocarcinoma)		• [Pd(L^2^)_2_]: apoptosis, increased sub-G1 cell cycle population, induced mitochondrial dysfunction • ↑ ROS levels, mitochondrial membrane depolarization, cytochrome *c* release, caspase overexpression, Bax overexpression, Bcl-2 under-expression	(26)
	[Pd(sac)_2_(dppm)], [Pd(sac)_2_(dppe)], [Pd(dppm)_2_](sac)_2_, [Pd(dppe)_2_](sac)_2_ (compared to Pt(II) analogues and cDDP); L: saccharinate (sac), 1,1-bis(diphenyl phosphino)methane (dppm),1,2-bis(diphenyl phosphino)ethane (dppe)	MCF-7 (breast cancer), A549 (lung cancer), DU145, HCT116 (colon cancer), BEAS-2B (bronchial epithelial)		•* Cationic Pd-dppm and Pd-dppe: more active in all cells* •* Neutral/cationic Pd-dppm complexes: apoptosis through caspases-3/7 activity, B-phase cell arrest*, ↑*ROS levels*	(27)
**Polynuclear**	Pd_2_Spm [compared to Pt(II) analogue and cDDP]; L: spermine (Spm)	A2780, A2780/CP (ovarian cancer, CP: cDDP resistant)		• Affects SMO, arginase 2, down-regulates NRF-2 • ↓ biogenic PAs putrescine, spermidine, spermine	(28)
	Pd_2_[S_(−)_C^2^, N-dmpa]_2_ (μ-dppe)Cl_2_(compared to cDDP); L: *N,N-*dimethyl-1-phenethyl-amine (dmpa), 1,2-ethanebis(diphenyl phosphine) (dppe)	B16F10-Nex2 (murine melanoma) (more lines for other studies and comparison to *in vivo*)		• Reacts with protein thiol groups in mitochondrial membrane, changes membrane potential, induces Bax translocation into mitochondria • Mitochondria and ER damage (changed Ca^2+^, ATP, endonucleases levels) • Caspases activation	(29)
	Pd_2_Spm, Pd_2_BENSpm (compared to Pt_2_CPENSpm); L: spermine (Spm), *N* ^1^,*N* ^11^-bis(ethyl) norspermine (BENSpm), *N* ^1^-cyclo-propylmethyl-*N* ^11^-ethylnorspermine (CPENSpm)	MCF-10A (normal-like breast epithelial), JIMT-1, L56BR-C1 (breast cancer)		• L56Br-C1: Pd_2_BENSpm decreased PAs levels, increased SSAT activity • Pd_2_BENSpm is selectively cytotoxic for breast cancer cells, with low toxicity for non-neoplastic MCF-10A cells • Pd_2_Spm: ↓GSH levels (L56Br-C1 most sensitive)	(10)
	Pd_2_Spm (compared to Pt analogues and cDDP); L: spermine (Spm)	MDA-MB-231 (breast cancer)		• Pd_2_Spm and Pt_2_Spm: distinct cytotoxicity pathways, ↑ lipids, changes in DNA/protein structures • ↑ Thymine; ↓ adenine, cytosine, guanine, deoxyguanine, deoxyribose	(30)
	Pd_2_Spm (compared to cDDP; single and Dox/Mtx combined administration); L: spermine (Spm)	MG-63 (osteosarcoma), HOb (osteoblasts)		* *Pd2Spm* alone: no apoptosis, less and reversible changes in MG-63 (↓GSH, inositol, ↑hypoxanthine, ↓UXP, ↑PC), changes amino acid levels in HOb * *Combined Pd2Spm: apoptosis, changes in lipids, choline compounds, amino acids, nucleotides, GSH, inositol, hypoxanthine (antioxidant defense)*	(31)
***In vivo* studies**					
**Mononuclear**	[Pd(sac)(terpy)](sac)•4H_2_O (compared to cDDP and paclitaxel); L: saccharinate (sac), 2,2’:6’,2’’-terpyridine (terpy)	Balb/c mice subcutaneously injected with Ehrlich ascites carcinoma (EAC) cells (comparison to *in vitro)*		* Mild-to-low toxic profile (evaluated through animal weight), comparable to paclitaxel and less toxic than with cDDP (delays cancer growth)	(21)
	[Pd(acac)_2_] (compared to cDDP); L: bisacetylacetonate (acac)	H460 (lung cancer) xenograft mouse model (Balb/c) (comparison to *in vitro)*		* Significant toxic profile but lower than with cDDP (better antitumor activity)	(23)
**Polynucl.**	Pd_2_ [S_(−)_C^2^, N-dmpa]_2_ (μ-dppe)Cl_2_ (compared to cDDP); L: *N,N-*dimethyl-1-phenethyl-amine (dmpa),1,2-ethanebis (diphenylphosphine) (dppe)	Wistar rats and B16F10-Nex2 (melanoma) xenograft mice (C57Bl/6) (comparison to *in vitro)*		* Wistar rats: disturbed permeabilization of mitochondria membranes * Xenograft mice: reduced number of nodules, anti-metastatic effect	(29)

ATP, adenosine triphosphate; Bax, Bcl-2-associated X protein; Bcl-2, B-cell lymphoma 2; CAT, catalase; cDDP, cisplatin; CHOP, growth arrest and DNA damage-inducible gene 153 (GADD153); Dox, docetaxel; ER, endoplasmic reticulum; JNK, Jun N-terminal kinase; GPX, glutathione peroxidase; GSH, glutathione (reduced); GST, glutathione transferase; GSTp1, glutathione S-transferase pi; IRE1, inositol-requiring enzyme 1; PARP, poly(ADP-ribose) polymerase (DNA-repair enzyme); PA, polyamine; PLS-DA, partial least squares—discriminant analysis; PC, phosphocholine; PTK, protein tyrosine kinase; MTX, methotrexate; n/a, not applicable; NRF-2, nuclear factor erythroid 2–related factor 2; ROS, reactive oxygen species; SMO, spermine oxidase; SSAT, spermidine/spermine N^1^-acetyltransferase (SSAT-1).

### Mononuclear Pd(II) Complexes

An early study tested curcumin, the main yellow pigment of turmeric, as a ligand, together with 4,4′-dinonyl-2,2′-bipyridine (bipy) (19) (**Table 1**). Curcumin is believed to play several beneficial roles on human health, *e.g.*, anti-inflammatory and antioxidant. The action of the [(bipy)Pd(Pcurc)][CF_3_SO_3_] complex on several human prostate cancer lines revealed induction of apoptosis (with caspase-3 activation), associated with production of reactive oxygen species (ROS) and mitochondrial membrane depolarization. Interestingly, the complex did not seem to affect DNA structure or the activity of the DNA-repair enzyme PARP, contrary to the free curcumin ligand. This demonstrates the importance of ligand complexation, which in the case of [(bipy)Pd(Pcurc)][CF_3_SO_3_], seems to determine intracellular mechanistic action. Also tested on prostate cancer cells (this time of the PC-3 cell line), a luminescent Pd(II) complex, [Pd(L)Cl], with acyclic tridentate ligand quinoline-2-carboxaldehyde-2-pyridylhydrazone, also revealed caspase-3 activation as indicative of cell apoptosis (20). These effects were associated with cell cycle modulation indicated G2/M phase arrest and increased cytochrome *c* expression, indicative of a deviant mitochondrial pathway.

Breast cancer cell lines have been the target of many studies testing the anti-cancer action of Pd(II)-based complexes. An earlier report addressed the action of a Pd(II) complex with saccharinate (sac) and 2,2’:6’,2’’-terpyridine (terpy), [Pd(sac)(terpy)](sac)•4H_2_O, on two metabolically distinct breast cancer cell lines: estrogen-receptor-positive MCF-7 and triple-negative MDA-MB-231 (metastatic) (21). The authors report, for the first time to their knowledge, the increased expression of specific genes and proteins related to cell death receptors. An increment in the protein corresponding to the DR5 cell death receptor gene was interpreted as indicative that activation of cell death receptors by the Pd(II) complex is the main mechanism of apoptosis induction. However, *in vitro* results revealed different behaviors for the two cell lines, with MCF-7 cells becoming more actively apoptotic than MDA-MB-231 cells. The latter responded to the complex with reduced ability for tubules formation (and, hence, formation of cell networks), which may reflect an ability of the Pd(II) complex to reduce metastasis formation in triple-negative breast cancer cells. A few years later (25), the same authors published a full biochemical and proteomic study of the same complex on MDA-MB-231 cells, compared to human lung adenocarcinoma (A549) and epithelial cervical cancer (HeLa) cell lines. About 30 proteins were identified with altered expression levels in MDA-MB-231 cells, as a result of complex exposure. These proteins were suggested to impact on multiple cellular processes, *e.g.*, energy metabolism, double strand break repair mechanisms, membrane trafficking, protein degradation and apoptosis (**Table 1**). This report on proteomics and subsequent pathway analysis for data interpretation illustrates the large levels of complex information retrievable from the use of untargeted omic approaches.

**Figure 1:**
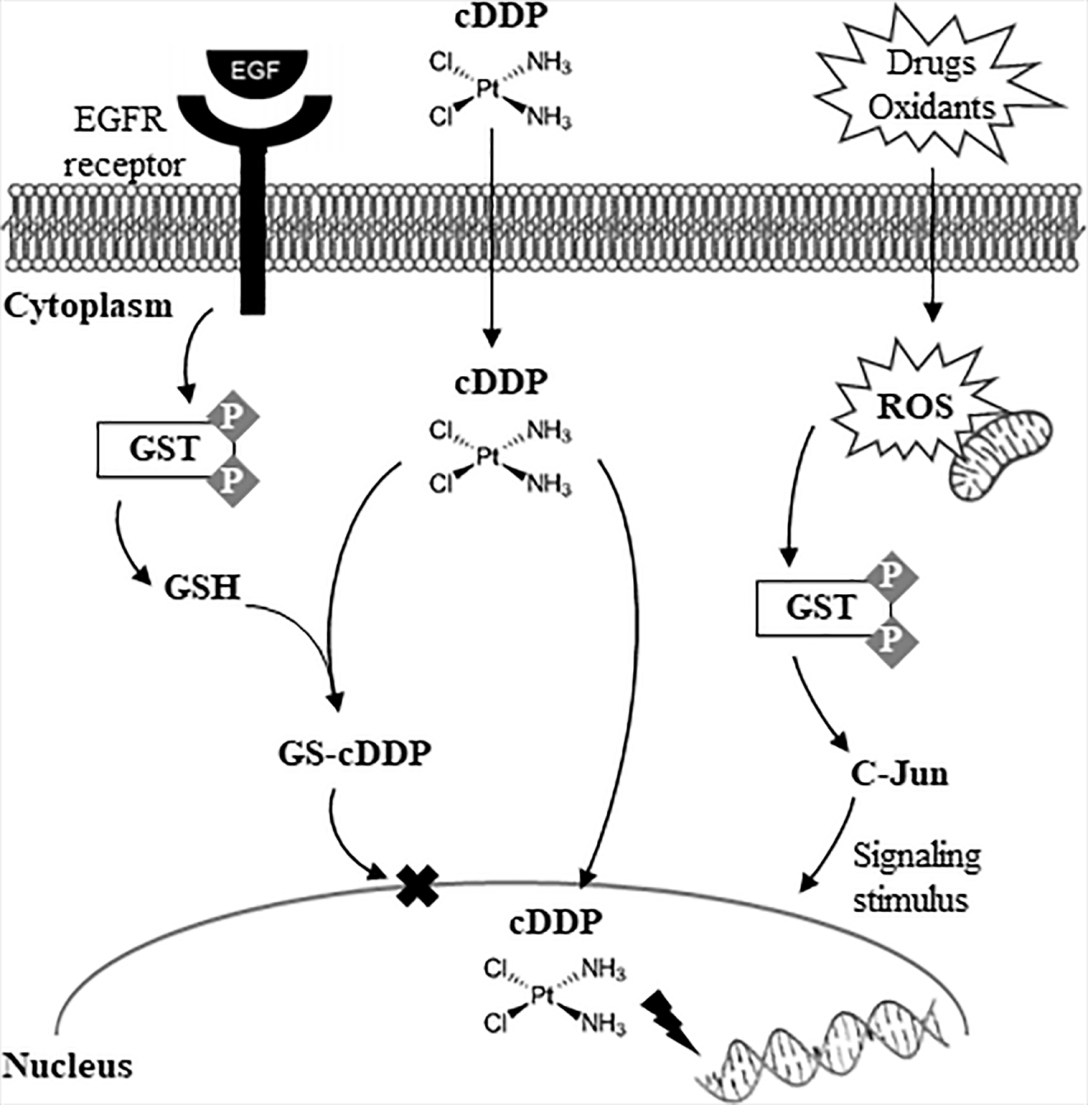
The coordination mechanism of Pt(II)-derived drug cDDP (cisplatin) with GSH and the consequent effect of GST-mediated drug resistance. Adapted from reference (24).

The epithelian cervical cancer cell line HeLa was also used to test the Pd(MCO)_2_ complex (with ligand 2-cyano-2-isonitroso-*N-*morpholylacetamide), compared to Pt(MCO)_2_ (22). Results showed that the complexes induced no change in mitochondrial metabolism, although overall results suggested different metabolic impacts of the Pd/Pt(MCO)_2_ complexes, compared to those of cisplatin family. This was interpreted as a possible circumvention of the negative side effects usually associated with cisplatin. A subsequent paper has described the effect of [Pd(acac)_2_] (acac: bisacetylacetonate), a Pd-O complex, on several cancer cell lines and, in particular, on human non-small-cell lung cancer H460 cells (23), to investigate the mechanism through which apoptosis was induced. The authors concluded that the apoptotic mechanism did not seem to relate to DNA but, rather, was mediated by endoplasmic reticulum (ER) stress pathway. ER stress increase was reflected by increased expression of the CHOP protein (associated to growth arrest and DNA damage-inducible gene 153), known to play a key role on the ER pathway. Since the ER regulates intracellular Ca^2+^ levels, the lower Ca^2+^ levels observed upon treatment with the Pd(II) complex were interpreted as reflecting ER stress, together with accumulation of misfolded protein in ER. The authors suggested that this apoptotic mechanism could be explained by the Pd-O nature of the complex (instead of Pd-N), which may prevent interaction with S-ligand- or N-ligand-containing molecules (*e.g.*, DNA). In the same year, a comprehensive study of Pd(diethyl-dithiocarbamate)_2_, compared to its Pt(II) analogue and other symmetrical mononuclear Pt(II) and Ni(II) complexes (24), on human leukemia cells (K562 cell line) revealed detailed information on glutathione utilization, antioxidant mechanisms and kinase signaling, aiming at investigating the impact on cellular defense mechanisms. **Figure 1** shows part of the different cellular defense mechanisms believed to be triggered by cisplatin, for instance, and correlate to development of drug resistance. In this study of Pd(diethyl-dithiocarbamate)_2_, the activity of several enzymes was measured including for glutathione-transferase (GST) enzymes (associated to drug resistance and cancer development) and protein tyrosine kinase (PTK) (**Table 1**). Results showed that the extent of antioxidant defense is strongly dependent on the nature of the metal complex, Pd(diethyl-dithiocarbamate)_2_ exhibiting favorable antioxidant characteristics, in tandem with good antitumor performance. A later systematic study of four Pd(II)[bis-2-(arylazo)phenolates] on human lung carcinoma (A549), cervical carcinoma (HeLa) and teratocarcinoma (PA-1) cell lines (26) helped to single out one of the complexes as the most effective in inducing apoptosis in all cell lines, while causing mitochondrial dysfunction affecting several enzymes and raising ROS levels (**Table 1**).

Following on earlier studies of Pd(II) complexes with saccharinate as a ligand (21, 25), a more recent report by the same group further exploited the promise of Pd(II)/Pt(II) complexes with saccharinate, now combined with diphoshine ligands (27). A series of neutral and cationic Pd(II) saccharinate complexes with 1,1-bis(diphenyl-phosphino)methane (dppm) or 1,2-bis(diphenyl diphosphino)ethane (dppe) were synthesized and subsequently tested on several human cancer cell lines: breast (MCF-7), lung (A549), prostate (DU145), colon (HCT116), as well as on healthy bronchial epithelial cells (BEAS-2B). This systematic report enabled the identification of cationic Pd-dppm and Pd-dppe complexes as promising as potential anti-cancer agents, while the former (Pd-dppm, either of neutral or cationic nature) seemed to cause clearer apoptosis, accompanied by cell arrest at the S-phase stage (DNA synthesis) and production of high levels of ROS.

### Polynuclear Pd(II) Complexes

All studies listed for polynuclear Pd(II) complexes relate to bidentate chelates and most refer to complexes bearing biogenic polyamines (PAs) (**Table 1**). The exception relates to a study of a biphosphinic cyclopalladated complex with *N,N-*dimethyl-1-phenethyl-amine (dmpa) and 1,2-ethanebis(diphenylphosphine) (dppe) as ligands (29) (**Table 1**). This complex was tested *in vitro* on murine melanoma cell lines. The results indicated mitochondrial dysfunction, in tandem with increased cytosolic Ca^2+^ levels and decreased ATP levels, as well as activation of caspase enzymes, triggering apoptosis.

In relation to biogenic PA complexes, the Pd_2_Spermine (Spm) and Pt_2_Spm bidentate complexes have been compared early on (28) as to their effects on the expression of genes involved in the polyamine pathway. This metabolic pathway has been recognized as an important target for therapeutic strategies since PA are required for cell proliferation and are, thus, usually found elevated in tumor tissues (28). The Pd_2_Spm complex was found to induce a similar effect on several polyamine pathway enzymes as Pt_2_Spm, both compounds inducing distinguishing enzymatic profiles compared to cisplatin. Interestingly, Pd_2_Spm induced significant declines in biogenic PA levels, whereas the Pt(II) analogue did not (28), thus pinpointing Pd(II)/PA complexes as promising agents in therapeutic strategies. Pd_2_Spm was subsequently compared to Pd_2_BENSpm [with PA analogue *N* ^1^,*N* ^11^-bis(ethyl)norspermine as ligand] and Pt_2_CPENSpm (with PA analogue *N* ^1^-cyclo-propylmethyl-*N* ^11^-ethylnorspermine), as well as with the free ligands BENSpm and CPENSpm) (10), in relation to its action against both normal (MCF-10A) and breast cancer (JIMT-1, L56BR-C1) cell lines (**Table 1**). Comparison with normal cell lines is important to evaluate the action of potential anti-cancer drugs on healthy tissue, thus helping to identify effects related to possible side effects. L56BR-C1 cells seemed to be significantly sensitive to Pd(II) complexes, Pd_2_BENSpm inducing decreased PA levels and increased spermidine/spermine *N^1^-*acetyltransferase (SSAT) activity, whereas Pd_2_Spm induced lower glutathione (GSH) levels (including in other cell lines studied). In addition, Pd_2_BENSpm was found to elicit quite low toxicity toward non-tumorigenic breast epithelial cells (IC_50_ = 34.2 µM *vs.* 7.3 and 0.4 µM for breast cancer cell lines), which is a promising result supporting its possible use as an anticancer drug. A later study (30) compared the same Pd_2_Spm and Pt_2_Spm complexes using vibrational spectroscopy and unsupervised multivariate analysis to identify changes in several cellular components. The study identified changes in the levels of cellular lipids and several nitrogen bases, as well as in DNA and protein structure (conformational changes or proteolysis), as resulting from exposure to the spermine-based complexes (**Table 1**). A more recent study tested Pd_2_Spm on osteosarcoma cells and osteoblasts, compared to cisplatin, using untargeted high-resolution-magic-angle-spinning (HRMAS) nuclear magnetic resonance (NMR) spectroscopy metabolomics (31). HRMAS NMR enables the direct analysis of cells, thus providing information simultaneously on polar and apolar components. The Pd(II) complex alone was seen to affect the metabolome of osteoblasts more extensively than that of cancer cells, evidencing no indication of apoptosis, contrary to cisplatin alone. However, when combined with doxorubicin and methotrexate, the Pd(II) complex impacted more strongly on cancer cells, in a similar way to the equivalent cisplatin-based combination: apoptosis was induced and strong variations were noted in lipids, choline compounds (cell membrane metabolism), several amino acids, nucleotides, and compounds related to antioxidative defense mechanisms (GSH and, possibly, inositol and hypoxanthine). This showed that an untargeted metabolomics strategy can unveil a large number of *a priori* unknown responsive metabolites (and pathways). In this case, this approach revealed that Pd_2_Spm can impact differently on cell metabolism (of both cancer and healthy cells), depending on whether it is administered alone or combined with other drugs. In fact, the promise of such strategies in revealing hidden features of cellular response to metal complexes with potential anti-cancer action has been recently reviewed, giving particular emphasis to NMR as the chosen analytical technique (32).

## *In Vivo* Studies of the Metabolic Impact of Pd(Ii) Complexes

*In vivo* studies are still few in number and the existing reports tend to describe indirect features of altered metabolism, in comparison with *in vitro* observations (21, 23, 29). Three different Pd(II) complexes have been studied *in vivo* in murine animal models (all in tandem with *in vitro* studies) (**Table 1**): [Pd(sac)(terpy)](sac)•4H_2_O, [Pd(acac)_2_] and Pd_2_ [S_(−)_C^2^, N-dmpa]_2_ (μ-dppe)Cl_2_. Reports generally indicate that these Pd(II) complexes induce a lesser toxic profile than cisplatin on animals, as evaluated by animal weight, while performing more effectively in terms of antitumor activity. However, to our knowledge, no further specific metabolic information has been reported on either toxicity or anticancer activity of Pd(II) complexes in an *in vivo* context, thus clearly unveiling a niche of research which would be interesting to pursue.

## Discussion

This mini-review has shown that most of the existing knowledge on the effects of Pd(II) complexes with potential anticancer activity on cell lines has been based on the important understanding of the mechanisms of induced cell death and antioxidant defense. The majority of studies have involved the measurement of changes at the gene and protein expression levels, usually adding measurements of ATP, ROS, and glutathione as markers of antioxidant mechanisms, and, in the specific cases of Pd(II) complexes with biogenic polyamines, the cellular levels of those compounds. Few reports have, so far, to the best of our knowledge, followed more untargeted approaches, although a tendency is noted for the use of omic sciences such as proteomics and metabolomics, involving analytical techniques such as mass spectrometry, vibrational spectroscopy and NMR spectroscopy. We propose that possible future developments in the context of metal complexes as anti-cancer agents, and particularly the promising Pd(II) complexes explored so far, may include the more extensive use of untargeted omic sciences, not only limited to study cancer cells but also including healthy/normal cells, in order to evaluate possible underlying metabolism deviations that may give rise to negative side effects. Furthermore, the issue of drug combination may follow naturally, based on studies that have shown how different the impacts of single and combined complexes may be. Finally, an increasing need of translational studies between *in vitro* and *in vivo* scenarios becomes clear, although the use of animal models is challenging and costly, with the added challenges of determination of drug doses of relevance and overall interpretation encompassing systemic response. In any case, metabolic players are, undoubtedly, an important part of cell/organism response and adaptation to therapeutic agents, their further study potentially contributing valuably for a more complete understanding of processes such as biotoxicity or development of drug resistance.

## Author Contributions

AG and TC conceived the scope of the manuscript. AG, TC, and MM were responsible for the literature search and selection, as well as for drafting the manuscript. AM and TC helped with final formatting of the manuscript, graphics and references. All authors contributed to the article and approved the submitted version.

## Funding

AG acknowledges funding from the CICECO-Aveiro Institute of Materials project, with references UIDB/50011/2020 & UIDP/50011/2020, financed by national funds through the FCT/MEC and when appropriate co-financed by FEDER under the PT2020 Partnership Agreement. MM acknowledges financial support from POCentro, COMPETE 2020, Portugal 2020 and European Community through the FEDER and the Portuguese Foundation for Science and Technology (UIDB/00070/2020). TC and AM are grateful to the Portuguese Foundation for Science and Technology (FCT) for grants SFRH/BD/145920/2019 and SFRH/BD/111576/2015, respectively.

## Conflict of Interest

The authors declare that the research was conducted in the absence of any commercial or financial relationships that could be construed as a potential conflict of interest.

## Supplementary Material

Supplementary Table 1 Compound names and chemical structures of Pd(II) complexes tested for some kind of metabolic effects, either *in vitro* or *in vivo*, as listed in **Table 1**. *: in these cases the structure of the complex could not be found. Compound chemical structures shown were drawn from scratch with basis on the references indicated.
