# Supplementary material for: Metabolic Aspects of Palladium(II) Potential Anti-Cancer Drugs
Source: Front Oncol. 2020 Oct 12;10:590970. doi: 10.3389/fonc.2020.590970 (PMC7586886; doi:10.3389/fonc.2020.590970)
Supplement: Supplementary Table 1 — Compound names and chemical structures of Pd(II) complexes tested for some kind of metabolic effects, either in vitro or in vivo, as listed in Table 1 . *: in these cases the structure of the complex could not be found. Compound chemical structures shown were drawn from scratch with basis on the references indicated. [file Table_1.docx]

**Table S1**. Compound names and chemical structures of Pd(II) complexes tested for some kind of metabolic effects, either *in vitro* or *in vivo*, as listed in Table 1. *: in these cases the structure of the complex could not be found. Compound chemical structures shown were drawn from scratch with basis on the references indicated.

|  | **Designation of Pd(II) complex (ligands identification)** | **Chemical structures of Pd(II) complexes** | | **Reference** |
| --- | --- | --- | --- | --- |
| **Mononuclear** | [(bipy)Pd(Pcurc)][CF_3_SO_3_]  (Pcurc, pure curcumin; bipy, 4,4′-dinonyl-2,2′-bipyridine) | 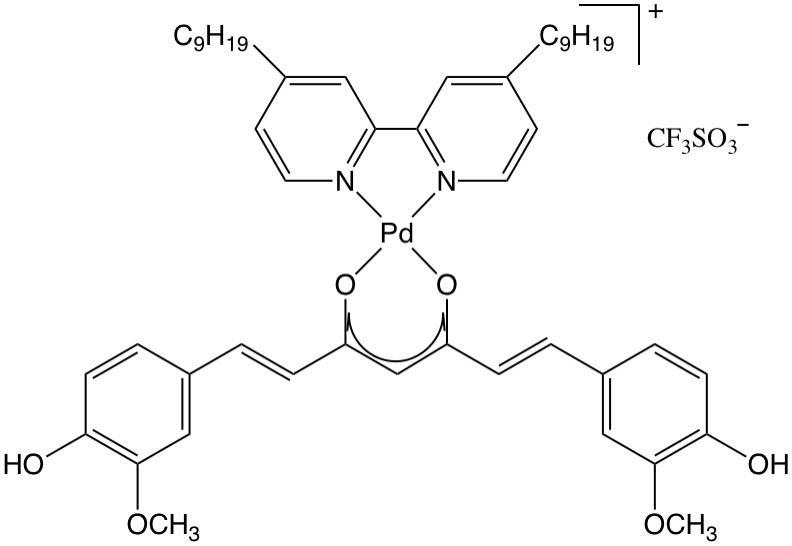 | | (19) |
|  | [Pd(L)Cl]  (HL, acyclic tridentate quinoline-2-carboxaldehyde-2-pyridylhydrazone) | 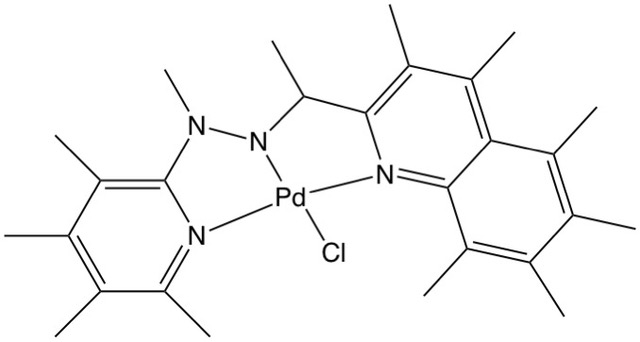 | | (20) |
|  | [Pd(sac)(terpy)](sac)•4H_2_O  (sac, saccharinate; terpy, 2,2’:6’,2’’-terpyridine) | 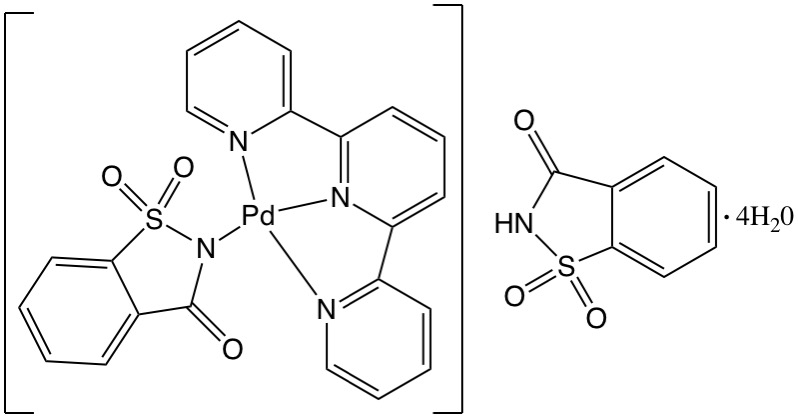 | | (21, 22) |
|  | Pd(MCO)_2_  (HMCO, 2-cyano-2-isonitroso-*N*-morpholylacetamide) | 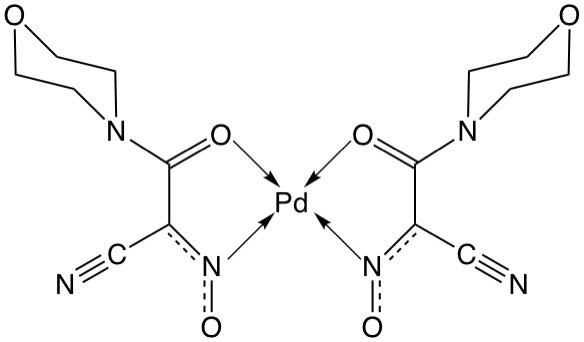 | | (23) |
|  | [Pd(acac)_2_]  (acac, bisacetylacetonate) | 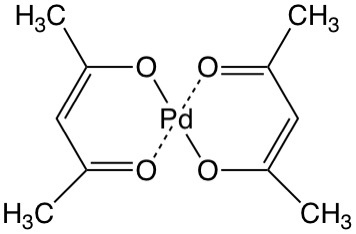 | | (24) |
|  | Pd(diethyl dithiocarbamate)_2_ | 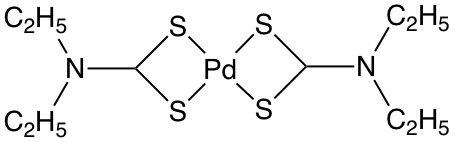 | | (25) |
|  | Pd_2_BENSpm  (BENSpm, *N*1,*N*11-bis(ethyl)norspermine) | **Ligand***  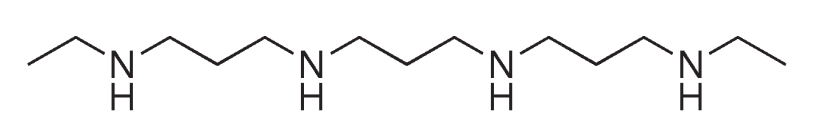 | | (10) |
|  | [Pd(L^1^)_2_], [Pd(L^2^)_2_], [Pd(L^3^)_2_], [Pd(L^4^)_2_]  (L^1^: R, H; R’=CH_3_)  (L^2^: R, CH_3_, H; R’=H)  (L^3^: R, CH_3_CH_2_; R’=H)  (L^4^: R, (CH_3_)_2_CH_2_; R’=H) | **Ligand***  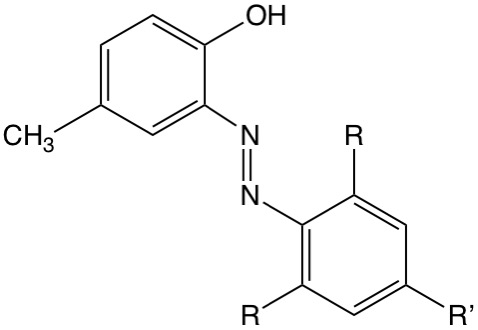 | | (26) |
|  | [Pd(sac)_2_(dppm)]  [Pd(sac)_2_(dppe)]  [Pd(dppm)_2_](sac)_2_  [Pd(dppe)_2_](sac)_2_  (sac, saccharinate; diphos, 1,1-bis(diphenyl phosphino)methane (dppm) or 1,2-bis(diphenyl phosphino)ethane (dppe)) | Neutral 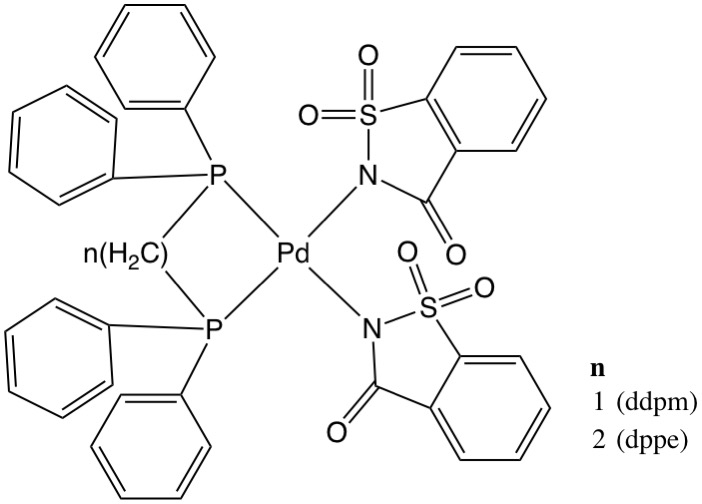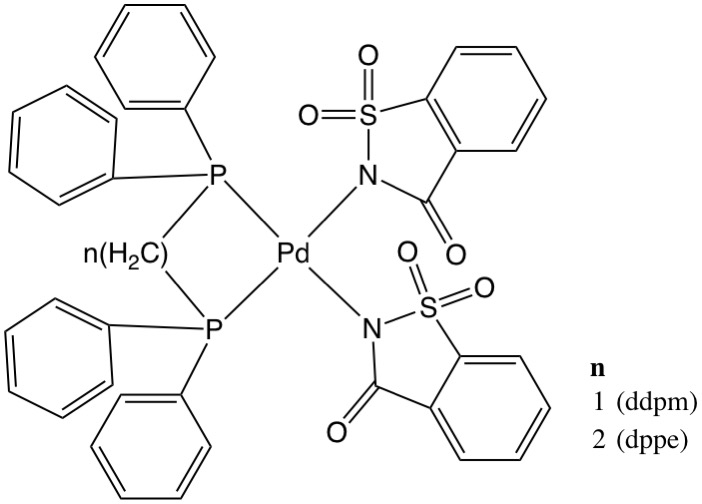 | Cationic 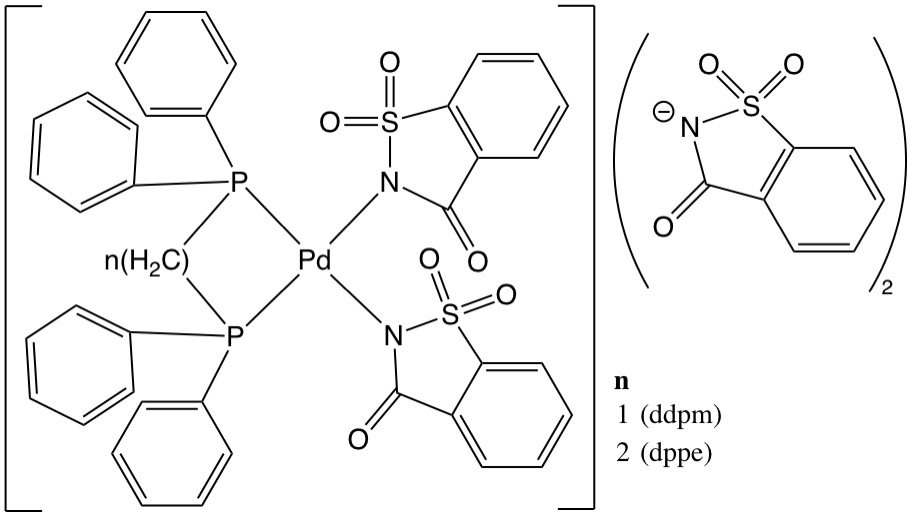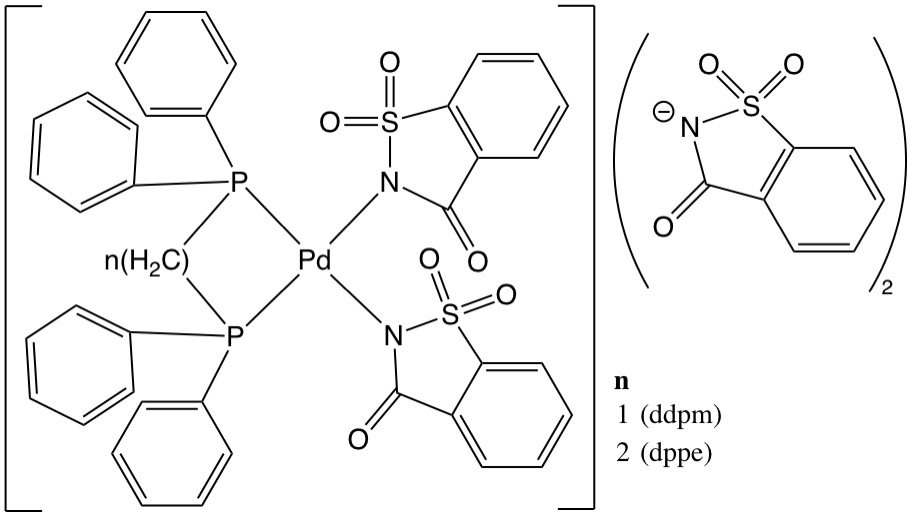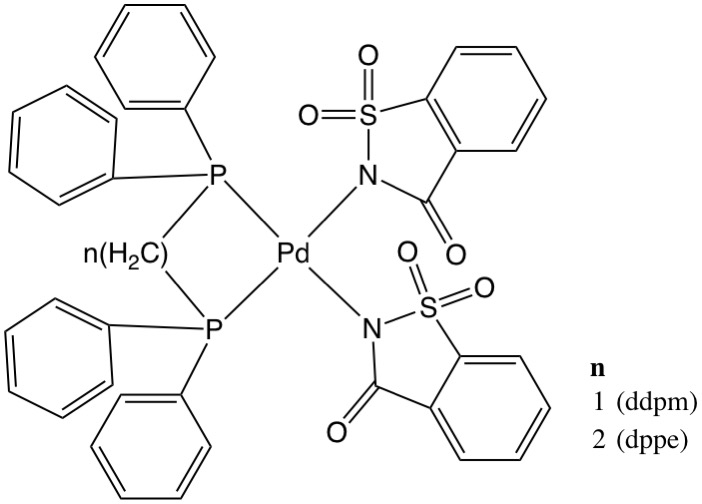 | (27) |
| **Polynuclear** | Pd_2_Spm  (Spm, spermine) | 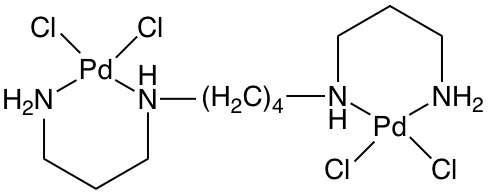 | | (28,30,31) |
|  | Pd_2_ [S_(-)_C^2^, N-dmpa]_2_ (μ-dppe)Cl_2_  (dmpa, *N*,*N*-dimethyl-1-phenethyl-amine; dppe, 1,2-ethanebis(diphenylphosphine)) | 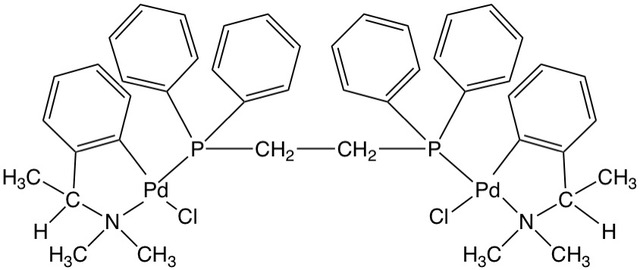 | | (29) |
